# (η^6^-Benzene)­chlorido­[2-(pyridin-2-yl)quinoline-κ^2^*N*,*N*′]ruthenium(II) tetra­fluorido­borate

**DOI:** 10.1107/S2414314624012409

**Published:** 2025-01-07

**Authors:** Manikandan Varadhan, Ibanpynhunlang Passi, Thangaraja Chinnathangavel, Venugopal Rajendiran

**Affiliations:** a Department of Chemistry School of Basic and Applied Sciences Central University of Tamil Nadu Thiruvarur 610 005 India; b Department of Chemistry North Eastern Hill University,Shillong 793 022 India; c Department of Chemistry, Anna University Regional Campus, Madurai 625 019, Tamil Nadu, India; Vienna University of Technology, Austria

**Keywords:** ruthenium(II), 2-(pyridin-2-yl)quinoline, η^6^-benzene, crystal structure

## Abstract

The title compound, [RuCl(C_6_H_6_)(C_14_H_10_N_2_)]BF_4_ or [Ru(η^6^-benzene)(*L*)Cl]^+^BF_4_^−^ [where *L* denotes the 2-(pyridin-2-yl)quinoline ligand], crystallizes in the monoclinic space group *P*2_1_/*c*. The coordination environment around Ru^II^ is best described as pseudo-octa­hedral, resembling the familiar half-sandwich ‘three-legged piano-stool’ shape. In the coordination sphere, the η^6^-binding benzene ligand coordinates with the central Ru^II^ atom occupying the ‘seat’ of the stool with a metal-to-centroid distance of 1.695 (17) Å, while the chelate ligand *L* coordinates with its N atoms and, together with the Cl ligand, defines the ‘legs’ of the stool. Apart from Coulombic forces, C—H⋯F and C—H⋯Cl hydrogen-bonding inter­actions consolidate the crystal packing.

## Abstract 2

The coordination environment around Ru^II^ is best described as pseudo-octa­hedral, resembling the familiar half-sandwich ‘three-legged piano-stool’ structure.

## Graphical abstract

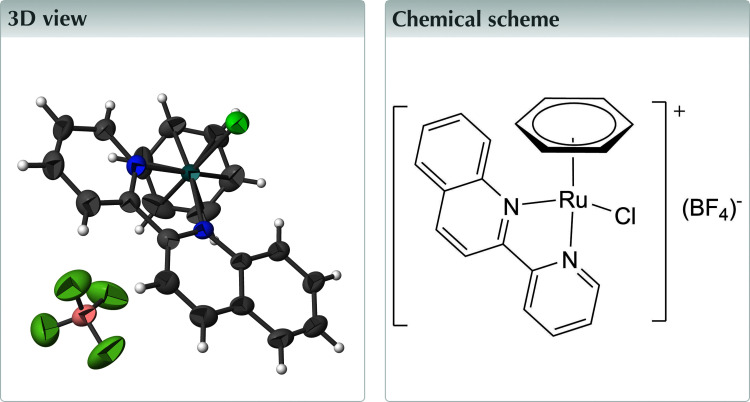

## Structure description

Ruthenium complexes exhibit a plethora of applications in the domains of medicinal chemistry (Casini & Pöthig, 2024 ▸; Rajendiran *et al.*, 2012 ▸; Chan *et al.*, 2017 ▸; Puckett & Barton, 2007 ▸), catalysis (Chavarot *et al.*, 2003 ▸; Ngo & Do, 2020 ▸; Hamelin *et al.*, 2007 ▸), and materials chemistry (Ryabov *et al.*, 2005 ▸; Huisman *et al.*, 2016 ▸; Vatsa & Padhi, 2021 ▸). Understanding their structural properties provides new insight into the design of novel ruthenium(II) complexes and predict their structure–activity relationships. In this context, the mononuclear mixed-ligand ruthenium(II) complex, [Ru(η^6^-benzene)(*L*)Cl]^+^BF_4_^−^ [where *L* is 2-(pyridin-2-yl)quinolone] has been synthesized and characterized by single-crystal X-ray analysis in the present work.

**Figure 1:**
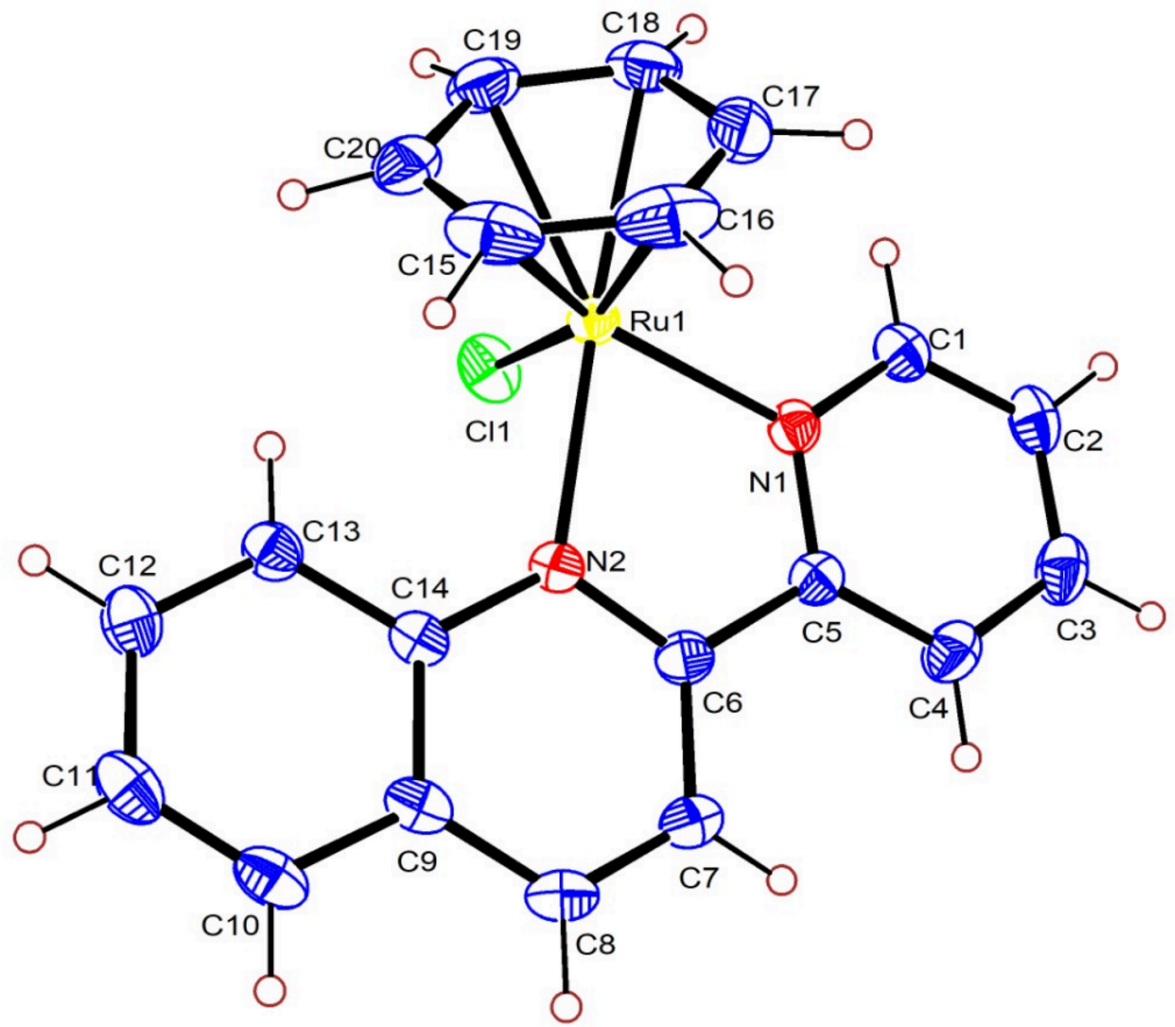
The mol­ecular structure of the complex cation of the title compound with displacement ellipsoids at the 50% probability level.

The distinctive half-sandwich, ‘three-legged piano-stool’ geometry of the complex cation of the title compound (Fig. 1 ▸) is characteristic of numerous η^6^-binding arene–ruth­enium(II) complexes (Khamrang *et al.*, 2016 ▸; Zamisa *et al.*, 2024 ▸). The ‘legs’ of the stool are defined by two σ-bonding N atoms from the chelating ligand *L* and the chlorido ligand, while the ‘seat’ is defined by the π-bonded benzene. The Ru^II^-to-benzene­(centroid) distance is 1.695 (17) Å and is comparable with other complexes containing *N*,*N*′-chelating ligands (Kelani *et al.*, 2023 ▸, 2024 ▸; Tsolis *et al.*, 2018 ▸). The Ru1—N1_py_ bond [2.086 (3) Å] is slightly shorter than the Ru1—N2_qn_ bond [2.147 (3) Å], revealing the pyridyl (py) N atom more firmly coordinates the central Ru^II^ atom than the quinolone (qn) N atom. The bidentate ligand has a bite angle of N1—Ru1—N2 = 76.42 (10)°. A similar type of coordination is observed in the crystal structure of [((2,2′-bipyrid­yl)(η^6^-*p*-cymene)iodido)­ruthenium(II)] hexa­fluorido­phosphate (Kelani *et al.*, 2023 ▸). The chlorido ligand bonds to the Ru^II^ atom with a distance of 2.3840 (9) Å. Except for the Ru—Cl bond, all other bonds are slightly longer than in the structure of the related complex [Ru(η^6^-*p*-cymene)*L*Cl]^+^(PF_6_)^−^ (Tsolis *et al.*, 2018 ▸).

**Figure 2:**
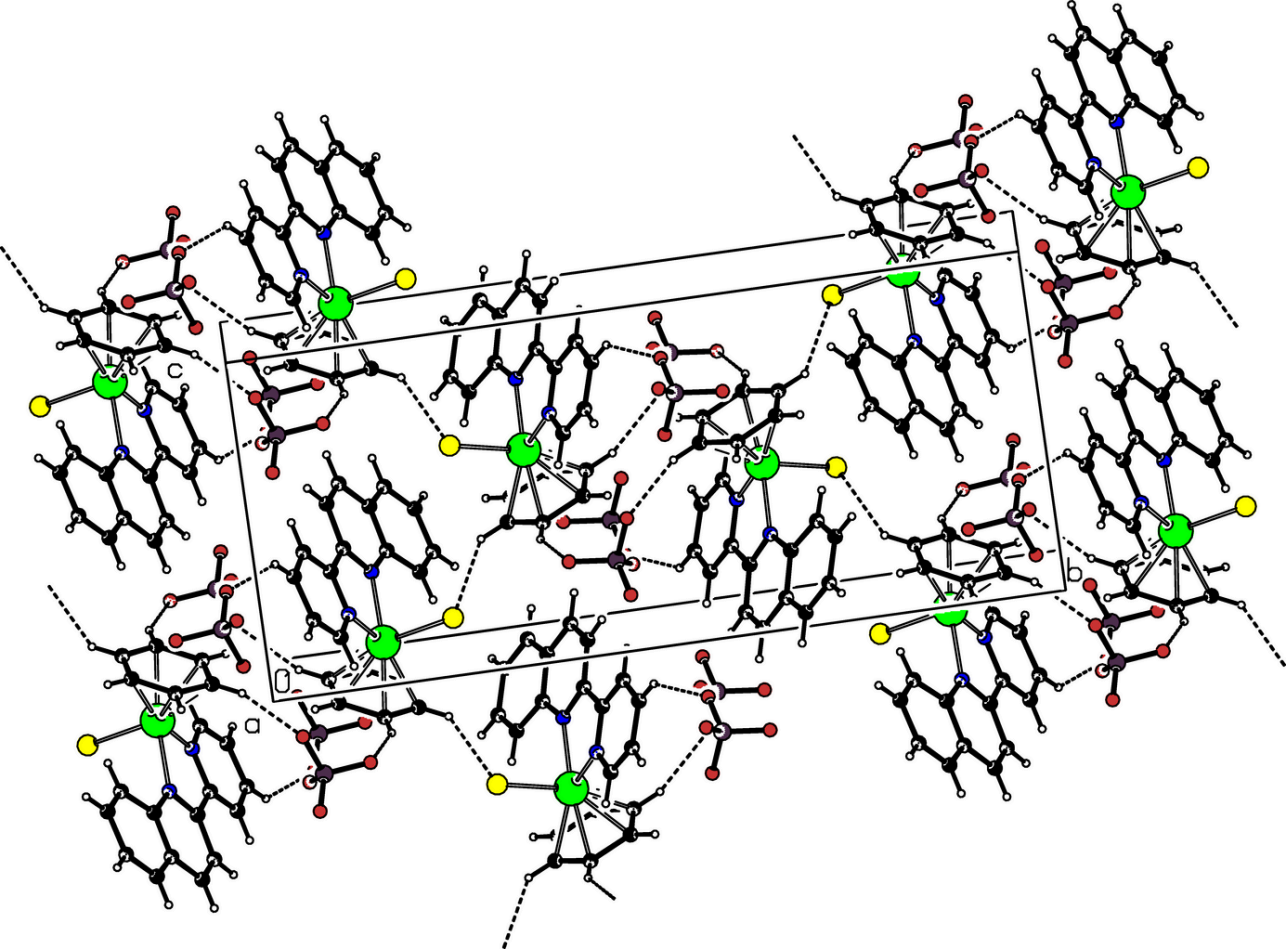
The crystal packing of the title compound. The Ru atoms are represented by green spheres, the Cl atoms by yellow spheres, and the F atoms by red spheres of arbitrary radii.

In the crystal, inter­molecular C—H⋯F and C—H⋯Cl hydrogen bonding (Table 1 ▸) plays a crucial role in the crystal packing (Fig. 2 ▸).

## Synthesis and crystallization

[Ru(*η^6^*-benzene)Cl]_2_ (0.12 g, 0.2 mmol) and *L* (2-(pyridin-2-yl)quinolone) (0.1 g, 0.4 mmol) were suspended in methanol (20 ml) and stirred at room temperature for 2 h. A solution of NaBF_4_ (200 mg, 0.60 mmol) in methanol (10 ml) was added to the initially orange solution, which changed color to yellow. After 24 h, the solution was evaporated, and the solid obtained was filtered off. The residue was washed with diethyl ether (40 ml) and dried under vacuum. The obtained product was recrystallized from a DCM:hexane mixture to give orange crystals. Yield: 65%.

## Refinement

Crystal data, data collection and structure refinement details are summarized in Table 2 ▸.

## Supplementary Material

Crystal structure: contains datablock(s) global, I. DOI: 10.1107/S2414314624012409/wm4225sup1.cif

Structure factors: contains datablock(s) I. DOI: 10.1107/S2414314624012409/wm4225Isup2.hkl

CCDC reference: 2407332

Additional supporting information:  crystallographic information; 3D view; checkCIF report

## Figures and Tables

**Table 1:** Hydrogen-bond geometry (Å, °)

*D*—H⋯*A*	*D*—H	H⋯*A*	*D*⋯*A*	*D*—H⋯*A*
C4—H4⋯F4^i^	0.93	2.48	3.375 (5)	162
C16—H16⋯F1	0.93	2.24	3.148 (6)	162
C19—H19⋯Cl1^ii^	0.93	2.65	3.429 (4)	142

Symmetry codes: (i) 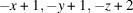; (ii) 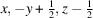.

**Table 2:** Experimental details

Crystal data	
Chemical formula	[RuCl(C_6_H_6_)(C_14_H_10_N_2_)]BF_4_
*M*_r_	507.68
Crystal system, space group	Monoclinic, *P*2_1_/*c*
Temperature (K)	293
*a*, *b*, *c* (Å)	8.4191 (3), 23.1476 (9), 9.9079 (3)
β (°)	94.709 (3)
*V* (Å^3^)	1924.35 (12)
*Z*	4
Radiation type	Mo *K*α
μ (mm^−1^)	1.00
Crystal size (mm)	0.65 × 0.50 × 0.41

Data collection	
Diffractometer	Agilent Xcalibur, Atlas, Gemini
Absorption correction	Multi-scan (*CrysAlis PRO*; Agilent, 2012 ▸)
*T*_min_, *T*_max_	0.507, 0.578
No. of measured, independent and observed [*I* > 2σ(*I*)] reflections	8112, 4311, 3815
*R*_int_	0.018
(sin θ/λ)_max_ (Å^−1^)	0.675

Refinement	
*R*[*F*^2^ > 2σ(*F*^2^)], *wR*(*F*^2^), *S*	0.040, 0.093, 1.13
No. of reflections	4311
No. of parameters	262
H-atom treatment	H-atom parameters constrained
Δρ_max_, Δρ_min_ (e Å^−3^)	0.92, −0.66

Computer programs: *CrysAlis PRO* (Agilent, 2012 ▸), *SHELXT* (Sheldrick, 2015*a* ▸), *SHELXL* (Sheldrick, 2015*b* ▸), *PLATON* (Spek, 2020 ▸) and *publCIF* (Westrip, 2010 ▸).

## full crystallographic data

### (η6-Benzene)chlorido[2-(pyridin-2-yl)quinoline-κ2N,N']\ ruthenium(II) tetrafluoridoborate Crystal data

**Table:**

[RuCl(C_6_H_6_)(C_14_H_10_N_2_)]BF_4_	*F*(000) = 1008
*M**_r_* = 507.68	*D*_x_ = 1.752 Mg m^−^^3^
Monoclinic, *P*2_1_/*c*	Mo *K*α radiation, λ = 0.71073 Å
*a* = 8.4191 (3) Å	Cell parameters from 4311 reflections
*b* = 23.1476 (9) Å	θ = 3.4–28.7°
*c* = 9.9079 (3) Å	µ = 1.00 mm^−^^1^
β = 94.709 (3)°	*T* = 293 K
*V* = 1924.35 (12) Å^3^	Block, orange
*Z* = 4	0.65 × 0.50 × 0.41 mm

### (η6-Benzene)chlorido[2-(pyridin-2-yl)quinoline-κ2N,N']\ ruthenium(II) tetrafluoridoborate Data collection

**Table:**

Agilent Xcalibur, Atlas, Gemini diffractometer	3815 reflections with *I* > 2σ(*I*)
Radiation source: fine-focus sealed tube	*R*_int_ = 0.018
ω scans	θ_max_ = 28.7°, θ_min_ = 3.4°
Absorption correction: multi-scan (CrysAlis PRO; Agilent, 2012)	*h* = −10→11
*T*_min_ = 0.507, *T*_max_ = 0.578	*k* = −29→31
8112 measured reflections	*l* = −13→7
4311 independent reflections	

### (η6-Benzene)chlorido[2-(pyridin-2-yl)quinoline-κ2N,N']\ ruthenium(II) tetrafluoridoborate Refinement

**Table:**

Refinement on *F*^2^	Primary atom site location: dual
Least-squares matrix: full	Hydrogen site location: inferred from neighbouring sites
*R*[*F*^2^ > 2σ(*F*^2^)] = 0.040	H-atom parameters constrained
*wR*(*F*^2^) = 0.093	*w* = 1/[σ^2^(*F*_o_^2^) + (0.0353*P*)^2^ + 2.2897*P*] where *P* = (*F*_o_^2^ + 2*F*_c_^2^)/3
*S* = 1.13	(Δ/σ)_max_ = 0.001
4311 reflections	Δρ_max_ = 0.92 e Å^−^^3^
262 parameters	Δρ_min_ = −0.66 e Å^−^^3^
0 restraints	

### (η6-Benzene)chlorido[2-(pyridin-2-yl)quinoline-κ2N,N']\ ruthenium(II) tetrafluoridoborate Special details

**Table:**

Geometry. All esds (except the esd in the dihedral angle between two l.s. planes) are estimated using the full covariance matrix. The cell esds are taken into account individually in the estimation of esds in distances, angles and torsion angles; correlations between esds in cell parameters are only used when they are defined by crystal symmetry. An approximate (isotropic) treatment of cell esds is used for estimating esds involving l.s. planes.

### (η6-Benzene)chlorido[2-(pyridin-2-yl)quinoline-κ2N,N']\ ruthenium(II) tetrafluoridoborate Fractional atomic coordinates and isotropic or equivalent isotropic displacement parameters (Å^2^)

**Table:**

	*x*	*y*	*z*	*U*_iso_*/*U*_eq_	
Ru1	0.36590 (3)	0.35365 (2)	0.55140 (2)	0.03087 (9)	
Cl1	0.48682 (11)	0.26281 (4)	0.60853 (11)	0.0510 (2)	
F2	0.0921 (4)	0.5390 (2)	0.8475 (3)	0.1168 (14)	
F3	0.1988 (4)	0.60983 (16)	0.7391 (5)	0.1201 (14)	
F4	0.3426 (3)	0.53126 (16)	0.7890 (3)	0.0961 (10)	
F1	0.1343 (5)	0.53076 (17)	0.6300 (3)	0.1116 (13)	
C19	0.3184 (6)	0.3211 (2)	0.3438 (4)	0.0585 (11)	
H19	0.342384	0.285320	0.307946	0.070*	
N2	0.2831 (3)	0.35589 (11)	0.7506 (3)	0.0313 (5)	
N1	0.5548 (3)	0.39139 (12)	0.6693 (3)	0.0353 (6)	
C14	0.1411 (4)	0.33333 (14)	0.7897 (3)	0.0335 (7)	
C18	0.4281 (5)	0.3648 (2)	0.3419 (4)	0.0543 (10)	
H18	0.524933	0.358972	0.305041	0.065*	
C9	0.0871 (4)	0.34711 (15)	0.9181 (3)	0.0395 (8)	
C11	−0.1533 (4)	0.29144 (18)	0.8644 (4)	0.0514 (10)	
H11	−0.251959	0.278213	0.887140	0.062*	
C20	0.1737 (5)	0.3286 (2)	0.3972 (4)	0.0630 (12)	
H20	0.100838	0.298376	0.395571	0.076*	
C2	0.8103 (4)	0.43585 (16)	0.6985 (4)	0.0473 (9)	
H2	0.905866	0.445181	0.662876	0.057*	
C12	−0.0967 (4)	0.27603 (17)	0.7396 (4)	0.0477 (9)	
H12	−0.157907	0.251981	0.680845	0.057*	
C5	0.5283 (4)	0.40623 (14)	0.7969 (3)	0.0353 (7)	
C3	0.7831 (4)	0.45260 (17)	0.8276 (4)	0.0500 (9)	
H3	0.858559	0.474045	0.879990	0.060*	
C8	0.1852 (5)	0.38125 (18)	1.0081 (4)	0.0493 (9)	
H8	0.151892	0.391143	1.092283	0.059*	
C1	0.6957 (4)	0.40530 (16)	0.6226 (4)	0.0430 (8)	
H1	0.715866	0.393750	0.535760	0.052*	
C13	0.0473 (4)	0.29590 (16)	0.7031 (4)	0.0402 (8)	
H13	0.083432	0.284658	0.620893	0.048*	
C10	−0.0633 (5)	0.32567 (17)	0.9513 (4)	0.0487 (9)	
H10	−0.100441	0.335336	1.034212	0.058*	
C6	0.3742 (4)	0.38674 (15)	0.8414 (3)	0.0368 (7)	
B1	0.1919 (5)	0.5506 (2)	0.7530 (4)	0.0497 (10)	
C15	0.1378 (5)	0.3804 (3)	0.4524 (4)	0.0678 (14)	
H15	0.041196	0.384661	0.490554	0.081*	
C16	0.2430 (7)	0.4273 (2)	0.4530 (4)	0.0717 (15)	
H16	0.216515	0.462854	0.488844	0.086*	
C7	0.3287 (5)	0.39968 (17)	0.9715 (4)	0.0462 (9)	
H7	0.396377	0.420705	1.031986	0.055*	
C4	0.6408 (4)	0.43679 (17)	0.8778 (4)	0.0480 (9)	
H4	0.620830	0.446693	0.965746	0.058*	
C17	0.3906 (6)	0.4190 (2)	0.3973 (4)	0.0636 (12)	
H17	0.463368	0.449282	0.397155	0.076*	

### (η6-Benzene)chlorido[2-(pyridin-2-yl)quinoline-κ2N,N']\ ruthenium(II) tetrafluoridoborate Atomic displacement parameters (Å^2^)

**Table:**

	*U*^11^	*U*^22^	*U*^33^	*U*^12^	*U*^13^	*U*^23^
Ru1	0.03032 (14)	0.03298 (15)	0.02952 (14)	0.00145 (11)	0.00382 (9)	−0.00114 (10)
Cl1	0.0498 (5)	0.0383 (5)	0.0662 (6)	0.0080 (4)	0.0128 (4)	0.0086 (4)
F2	0.083 (2)	0.191 (4)	0.082 (2)	−0.038 (2)	0.0366 (18)	−0.001 (2)
F3	0.093 (2)	0.080 (2)	0.189 (4)	−0.005 (2)	0.020 (3)	−0.010 (3)
F4	0.0582 (16)	0.126 (3)	0.104 (2)	0.0188 (18)	0.0070 (16)	0.032 (2)
F1	0.127 (3)	0.142 (3)	0.0637 (18)	0.040 (2)	−0.0099 (18)	−0.036 (2)
C19	0.081 (3)	0.059 (3)	0.0349 (18)	0.000 (2)	0.0039 (19)	−0.0128 (19)
N2	0.0278 (12)	0.0352 (14)	0.0311 (13)	0.0034 (11)	0.0027 (10)	0.0024 (11)
N1	0.0332 (14)	0.0358 (14)	0.0365 (14)	−0.0014 (12)	−0.0003 (11)	0.0022 (12)
C14	0.0297 (15)	0.0352 (16)	0.0363 (16)	0.0080 (13)	0.0057 (12)	0.0051 (13)
C18	0.047 (2)	0.084 (3)	0.0326 (17)	0.001 (2)	0.0095 (16)	0.0049 (19)
C9	0.0412 (18)	0.0405 (18)	0.0382 (17)	0.0108 (15)	0.0110 (14)	0.0080 (15)
C11	0.0379 (19)	0.055 (2)	0.063 (2)	0.0042 (18)	0.0152 (18)	0.014 (2)
C20	0.055 (2)	0.094 (4)	0.038 (2)	−0.020 (3)	−0.0052 (18)	−0.004 (2)
C2	0.0336 (18)	0.044 (2)	0.064 (2)	−0.0069 (16)	0.0032 (16)	0.0088 (18)
C12	0.0335 (17)	0.051 (2)	0.059 (2)	−0.0013 (16)	0.0058 (16)	0.0022 (18)
C5	0.0346 (16)	0.0353 (16)	0.0354 (16)	0.0029 (14)	−0.0009 (13)	0.0014 (13)
C3	0.0398 (19)	0.047 (2)	0.061 (2)	−0.0090 (17)	−0.0110 (17)	0.0007 (18)
C8	0.058 (2)	0.058 (2)	0.0327 (17)	0.005 (2)	0.0105 (16)	−0.0035 (17)
C1	0.0388 (18)	0.045 (2)	0.0455 (19)	−0.0018 (16)	0.0074 (15)	0.0033 (16)
C13	0.0346 (17)	0.0446 (19)	0.0418 (17)	0.0023 (15)	0.0057 (14)	0.0029 (15)
C10	0.048 (2)	0.051 (2)	0.051 (2)	0.0099 (18)	0.0216 (17)	0.0095 (18)
C6	0.0369 (17)	0.0393 (18)	0.0336 (16)	0.0032 (14)	0.0003 (13)	0.0012 (14)
B1	0.042 (2)	0.068 (3)	0.040 (2)	−0.004 (2)	0.0067 (17)	−0.002 (2)
C15	0.041 (2)	0.117 (4)	0.045 (2)	0.025 (3)	0.0020 (17)	0.019 (3)
C16	0.111 (4)	0.060 (3)	0.042 (2)	0.046 (3)	−0.004 (2)	0.008 (2)
C7	0.052 (2)	0.053 (2)	0.0335 (17)	−0.0034 (18)	0.0034 (15)	−0.0065 (16)
C4	0.047 (2)	0.051 (2)	0.0445 (19)	−0.0037 (18)	−0.0065 (16)	−0.0047 (17)
C17	0.086 (3)	0.055 (3)	0.047 (2)	−0.021 (2)	−0.013 (2)	0.020 (2)

### (η6-Benzene)chlorido[2-(pyridin-2-yl)quinoline-κ2N,N']\ ruthenium(II) tetrafluoridoborate Geometric parameters (Å, º)

**Table:**

Ru1—N1	2.086 (3)	C11—C12	1.407 (5)
Ru1—N2	2.147 (3)	C11—H11	0.9300
Ru1—C17	2.172 (4)	C20—C15	1.361 (8)
Ru1—C15	2.173 (4)	C20—H20	0.9300
Ru1—C16	2.183 (4)	C2—C1	1.371 (5)
Ru1—C19	2.196 (4)	C2—C3	1.373 (6)
Ru1—C18	2.197 (4)	C2—H2	0.9300
Ru1—C20	2.209 (4)	C12—C13	1.373 (5)
Ru1—Cl1	2.3840 (9)	C12—H12	0.9300
F2—B1	1.336 (5)	C5—C4	1.384 (5)
F3—B1	1.381 (6)	C5—C6	1.474 (5)
F4—B1	1.365 (5)	C3—C4	1.384 (5)
F1—B1	1.354 (5)	C3—H3	0.9300
C19—C18	1.371 (6)	C8—C7	1.359 (5)
C19—C20	1.379 (6)	C8—H8	0.9300
C19—H19	0.9300	C1—H1	0.9300
N2—C6	1.340 (4)	C13—H13	0.9300
N2—C14	1.388 (4)	C10—H10	0.9300
N1—C5	1.346 (4)	C6—C7	1.407 (5)
N1—C1	1.347 (4)	C15—C16	1.403 (7)
C14—C13	1.414 (5)	C15—H15	0.9300
C14—C9	1.423 (4)	C16—C17	1.413 (7)
C18—C17	1.416 (6)	C16—H16	0.9300
C18—H18	0.9300	C7—H7	0.9300
C9—C8	1.407 (5)	C4—H4	0.9300
C9—C10	1.423 (5)	C17—H17	0.9300
C11—C10	1.355 (6)		
			
N1—Ru1—N2	76.42 (10)	C15—C20—C19	119.8 (5)
N1—Ru1—C17	89.37 (14)	C15—C20—Ru1	70.5 (2)
N2—Ru1—C17	133.34 (16)	C19—C20—Ru1	71.2 (2)
N1—Ru1—C15	137.61 (19)	C15—C20—H20	120.1
N2—Ru1—C15	93.82 (13)	C19—C20—H20	120.1
C17—Ru1—C15	67.46 (19)	Ru1—C20—H20	130.9
N1—Ru1—C16	103.56 (17)	C1—C2—C3	119.5 (3)
N2—Ru1—C16	102.37 (14)	C1—C2—H2	120.2
C17—Ru1—C16	37.87 (19)	C3—C2—H2	120.2
C15—Ru1—C16	37.6 (2)	C13—C12—C11	121.1 (4)
N1—Ru1—C19	137.72 (15)	C13—C12—H12	119.5
N2—Ru1—C19	145.23 (14)	C11—C12—H12	119.5
C17—Ru1—C19	66.51 (17)	N1—C5—C4	121.0 (3)
C15—Ru1—C19	65.71 (18)	N1—C5—C6	114.9 (3)
C16—Ru1—C19	78.84 (17)	C4—C5—C6	124.1 (3)
N1—Ru1—C18	104.47 (14)	C2—C3—C4	118.4 (3)
N2—Ru1—C18	170.44 (14)	C2—C3—H3	120.8
C17—Ru1—C18	37.83 (17)	C4—C3—H3	120.8
C15—Ru1—C18	79.07 (16)	C7—C8—C9	119.7 (3)
C16—Ru1—C18	68.10 (17)	C7—C8—H8	120.1
C19—Ru1—C18	36.36 (16)	C9—C8—H8	120.1
N1—Ru1—C20	168.06 (15)	N1—C1—C2	122.4 (3)
N2—Ru1—C20	111.55 (14)	N1—C1—H1	118.8
C17—Ru1—C20	78.70 (17)	C2—C1—H1	118.8
C15—Ru1—C20	36.2 (2)	C12—C13—C14	120.5 (3)
C16—Ru1—C20	66.6 (2)	C12—C13—H13	119.8
C19—Ru1—C20	36.47 (17)	C14—C13—H13	119.8
C18—Ru1—C20	66.14 (16)	C11—C10—C9	121.3 (3)
N1—Ru1—Cl1	86.86 (8)	C11—C10—H10	119.4
N2—Ru1—Cl1	88.14 (7)	C9—C10—H10	119.4
C17—Ru1—Cl1	135.88 (15)	N2—C6—C7	123.0 (3)
C15—Ru1—Cl1	134.61 (17)	N2—C6—C5	115.6 (3)
C16—Ru1—Cl1	166.59 (13)	C7—C6—C5	121.4 (3)
C19—Ru1—Cl1	87.78 (13)	F2—B1—F1	111.3 (4)
C18—Ru1—Cl1	101.40 (12)	F2—B1—F4	112.1 (4)
C20—Ru1—Cl1	101.94 (15)	F1—B1—F4	112.4 (4)
C18—C19—C20	122.0 (4)	F2—B1—F3	107.6 (4)
C18—C19—Ru1	71.9 (2)	F1—B1—F3	105.2 (4)
C20—C19—Ru1	72.3 (2)	F4—B1—F3	107.8 (4)
C18—C19—H19	119.0	C20—C15—C16	121.5 (4)
C20—C19—H19	119.0	C20—C15—Ru1	73.4 (3)
Ru1—C19—H19	129.4	C16—C15—Ru1	71.6 (2)
C6—N2—C14	118.2 (3)	C20—C15—H15	119.2
C6—N2—Ru1	114.8 (2)	C16—C15—H15	119.2
C14—N2—Ru1	126.8 (2)	Ru1—C15—H15	128.1
C5—N1—C1	118.7 (3)	C15—C16—C17	117.9 (4)
C5—N1—Ru1	117.1 (2)	C15—C16—Ru1	70.8 (2)
C1—N1—Ru1	124.0 (2)	C17—C16—Ru1	70.6 (2)
N2—C14—C13	120.8 (3)	C15—C16—H16	121.0
N2—C14—C9	120.7 (3)	C17—C16—H16	121.0
C13—C14—C9	118.4 (3)	Ru1—C16—H16	129.8
C19—C18—C17	118.5 (4)	C8—C7—C6	119.5 (3)
C19—C18—Ru1	71.8 (2)	C8—C7—H7	120.2
C17—C18—Ru1	70.1 (2)	C6—C7—H7	120.2
C19—C18—H18	120.8	C3—C4—C5	120.0 (4)
C17—C18—H18	120.8	C3—C4—H4	120.0
Ru1—C18—H18	129.7	C5—C4—H4	120.0
C8—C9—C14	118.6 (3)	C16—C17—C18	120.2 (4)
C8—C9—C10	122.4 (3)	C16—C17—Ru1	71.5 (2)
C14—C9—C10	119.0 (3)	C18—C17—Ru1	72.1 (2)
C10—C11—C12	119.6 (3)	C16—C17—H17	119.9
C10—C11—H11	120.2	C18—C17—H17	119.9
C12—C11—H11	120.2	Ru1—C17—H17	128.8

### (η6-Benzene)chlorido[2-(pyridin-2-yl)quinoline-κ2N,N']\ ruthenium(II) tetrafluoridoborate Hydrogen-bond geometry (Å, º)

**Table:**

*D*—H···*A*	*D*—H	H···*A*	*D*···*A*	*D*—H···*A*
C4—H4···F4^i^	0.93	2.48	3.375 (5)	162
C16—H16···F1	0.93	2.24	3.148 (6)	162
C19—H19···Cl1^ii^	0.93	2.65	3.429 (4)	142

Symmetry codes: (i) −*x*+1, −*y*+1, −*z*+2; (ii) *x*, −*y*+1/2, *z*−1/2.
